# Robot-assisted thoracoscopic enucleation for a large esophageal leiomyoma: a case report

**DOI:** 10.1186/s40792-021-01212-9

**Published:** 2021-05-26

**Authors:** Kohei Kemuriyama, Satoru Motoyama, Yusuke Sato, Akiyuki Wakita, Yushi Nagaki, Hiromu Fujita, Ryohei Sasamori, Kazuhiro Imai, Masaki Aokawa, Yoshihiro Minamiya

**Affiliations:** 1 Esophageal Surgery, Akita University Hospital, 1-1-1 Hondo, Akita, 010-8543 Japan; 2 Thoracic Surgery, Akita University Graduate School of Medicine, Akita, Japan; 3 Comprehensive Cancer Control, Akita University Graduate School of Medicine, Akita, Japan; 4 Gastroenterology, Noshiro Kousei Medical Center, Noshiro, Japan

**Keywords:** Robot-assisted thoracoscopic surgery, Esophageal leiomyoma, Enucleation

## Abstract

**Background:**

Video-assisted thoracoscopic surgery (VATS) is being used to treat esophageal submucosal tumors (SMTs) all over the world. However, this technique is difficult when the tumor is large and located on the left side wall of the esophagus, within the upper mediastinum. This is because, with VATS, the surgical forceps have a limited range of motion. Robot-assisted thoracoscopic surgery (RATS) using the da Vinci surgical system may be extremely useful for enucleation of esophageal SMTs within the narrow upper mediastinum.

**Case presentation:**

A female in her thirties experiencing epigastric pain visited our hospital and was diagnosed with a large esophageal leiomyoma within the upper mediastinum. From its size (10 cm), it was judged to have malignant potential. We performed SMT enucleation using RATS with a da Vinci surgical system Xi. This was our second case using this system. The patient was placed in the left lateral position. Four da Vinci trocars (8 mm) were inserted into the 10th, 7th, 5th and 3rd intercostal spaces (ICS), and an assist port was added in the 5th ICS. We opened the superior mediastinal pleura cranially and caudally from the arch of the azygos vein and expanded the superior mediastinum after dividing the azygos vein. We made an incision in the muscular layer of the esophagus and, using a monopolar hook and monopolar scissors, enucleated the esophageal tumor in a protective manner so as not to damage its capsule or mucosa while applying appropriate robot-specific counter traction. We then sewed up the muscularis using 4–0 Vicryl, inserting the endoscope into the thoracic esophagus to substitute for a bougie. In addition, the pleura was sutured using barbed suture. The surgical procedure was straightforward and smooth. The patient was discharged on postoperative day 4 with no surgical complications. The tumor was definitively diagnosed pathologically from paraffin sections as a benign esophageal leiomyoma.

**Conclusions:**

RATS enables more delicate and precise esophageal SMT enucleation without surgical complications, though various challenges remain to be overcome.

**Supplementary Information:**

The online version contains supplementary material available at 10.1186/s40792-021-01212-9.

## Background

Esophageal leiomyoma is the most commonly occurring benign esophageal tumor and, thanks to advances in diagnostic imaging, it is often diagnosed at an asymptomatic stage, even in young adults [1]. If a definitive diagnosis of esophageal submucosal tumor (SMT) is made without pathological findings, we will follow up without treatment. However, if the tumor is large or growing, it may have malignant potential and is treated surgically, which enables us to make a pathological definitive diagnosis. In addition, surgery is indicated if there is chest discomfort, dysphagia, and/or upper abdominal pain. If the tumor is not confirmed to be malignant from intraoperative pathological examination of a frozen section, surgery most often involves denuclearization of the tumor followed by pathological examination of paraffin sections from the resected tumor. A decision is then made whether to perform an additional esophagectomy with lymph node dissection.

Video-assisted thoracoscopic surgery (VATS) is now widely used around the world for this type of tumor. However, use of VATS is difficult when the tumor is large and located on the left side wall of the esophagus, within the upper mediastinum. This is because VATS has only two-dimensional vision, and the thoracoscope and surgical forceps have limited ranges of motion. Use of robot-assisted thoracoscopic surgery (RATS) with the da Vinci surgical system has been increasing recently, as it provides magnified three-dimensional vision and finer motion that enables delicate tumor enucleation within the narrow thoracic cavity [2, 3]. There have been a number of reports of esophageal leiomyoma enucleation using VATS but use of RATS for esophageal SMT is not yet widespread, and there have been no reports its use for that purpose in Japan. Here, we report a case in which we used RATS to perform an SMT enucleation of an esophageal leiomyoma located in the upper and middle mediastinum, where it would be difficult to use VATS.

## Case presentation

**Fig. 1:**
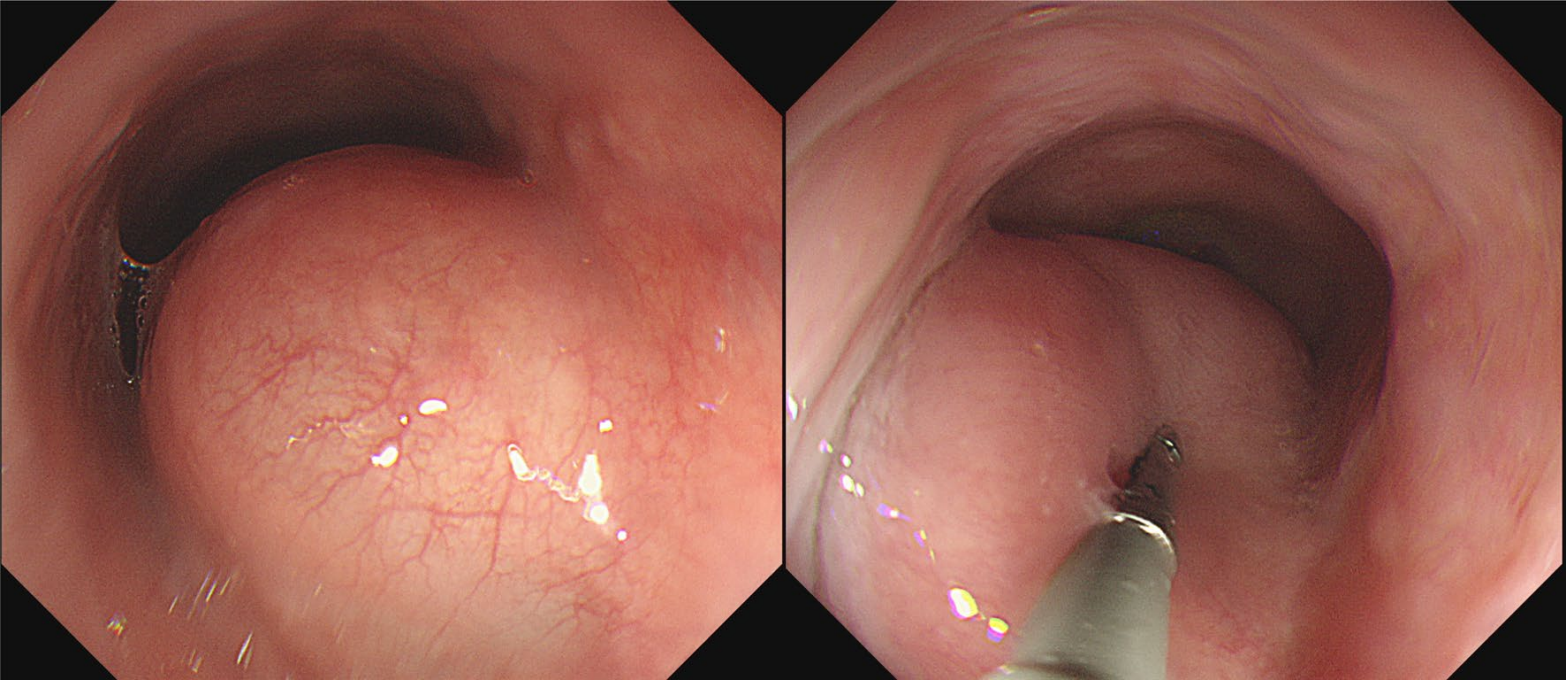
Upper gastrointestinal endoscopy showing a glossy and elastic soft esophageal submucosal tumor in upper and middle thoracic esophagus

**Fig. 2:**
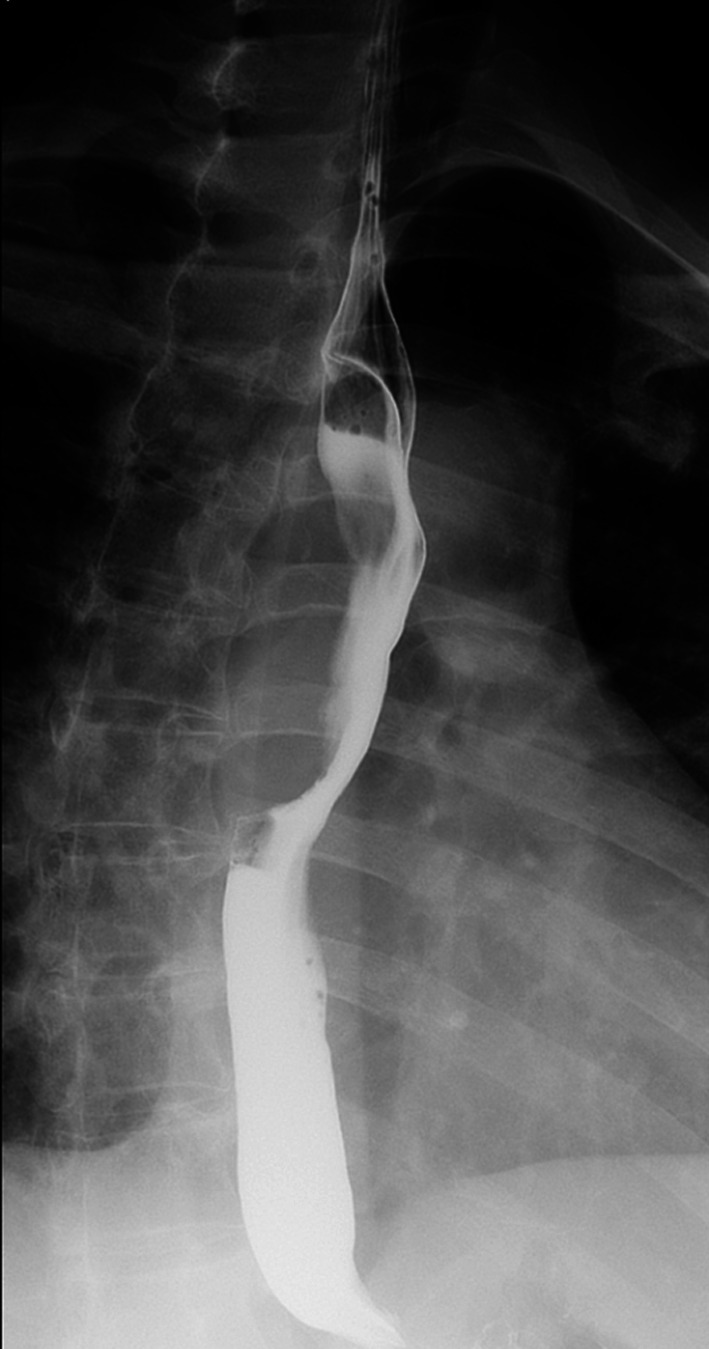
Esophagogram showing the lesion, which appears as a sharply demarcated, smooth, rounded, filling defect

**Fig. 3:**
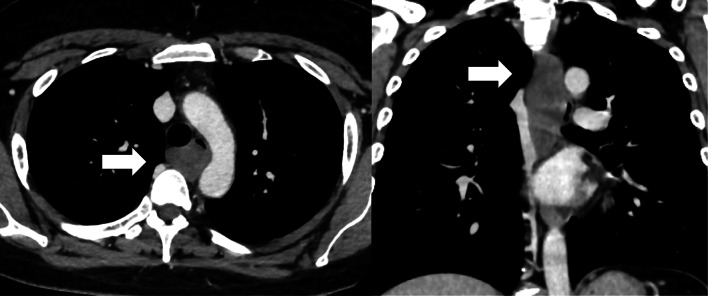
Enhanced computed tomography images

**Fig. 4:**
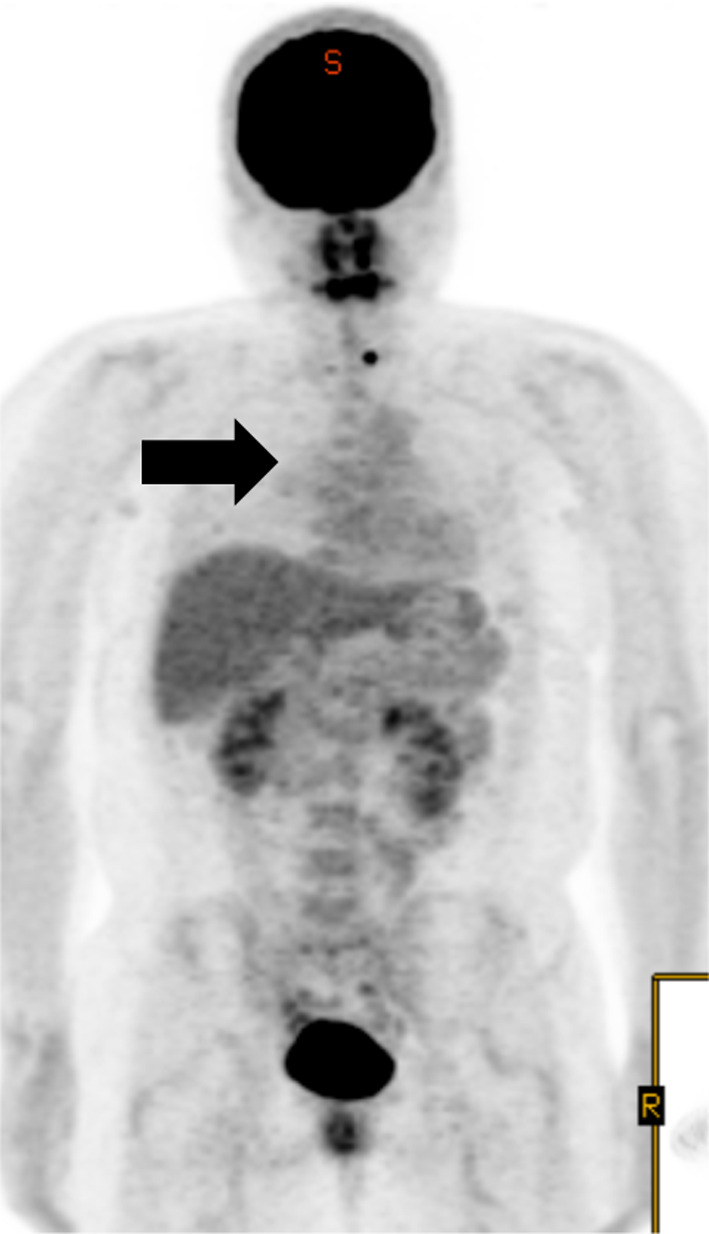
[^18^F] fluorodeoxyglucose-positron emission tomography showing no uptake into the tumor

A female in her thirties visited our hospital, because she was experiencing epigastric pain. Upper gastrointestinal endoscopy showed an esophageal SMT 10 cm in length about 20–30 cm from the incisors (Fig. 1). On an esophagogram the lesion appeared as a sharply demarcated, smooth, rounded, filling defect (Fig. 2). Enhanced computed tomography imaging of the chest showed a poor contrast effect mass in the upper-middle thoracic esophagus (Fig. 3). [^18^F] fluorodeoxyglucose-positron emission tomography detected no accumulation within the tumor (Fig. 4). Ultimately, after endoscopic ultrasound-fine needle aspiration, this esophageal SMT was diagnosed most likely to be a benign leiomyoma. However, from its size (10 cm) it was judged to have malignant potential. After sufficient explanation, the patient requested tumor enucleation to both treat her symptoms and for a definitive pathological diagnosis to rule out completely the possibility of malignancy.

We performed robot-assisted thoracoscopic esophageal SMT enucleation using a da Vinci Xi system, which was not covered by medical insurance. The patient was placed in the left lateral position under a combination of inhaled and intravenous anesthesia, and a double-lumen endotracheal tube was used for single-lung ventilation. The right arm was raised 60 degrees cranially to expose the right axillar fossa, then tilted 20 degrees cranially and 15 degrees ventrally. The da Vinci trocars were inserted into the 10th intercostal space (ICS), on a posterior axillary line (AL); the 7th ICS, on a middle AL; the 5th ICS, on a posterior AL; and the 3rd ICS on an anterior AL. An assist trocar with an air-sealed system was inserted into the 5th ICS on an anterior AL; it was used to maintain 8 mmHg positive pressure within the thoracic cavity.

**Fig. 5:**
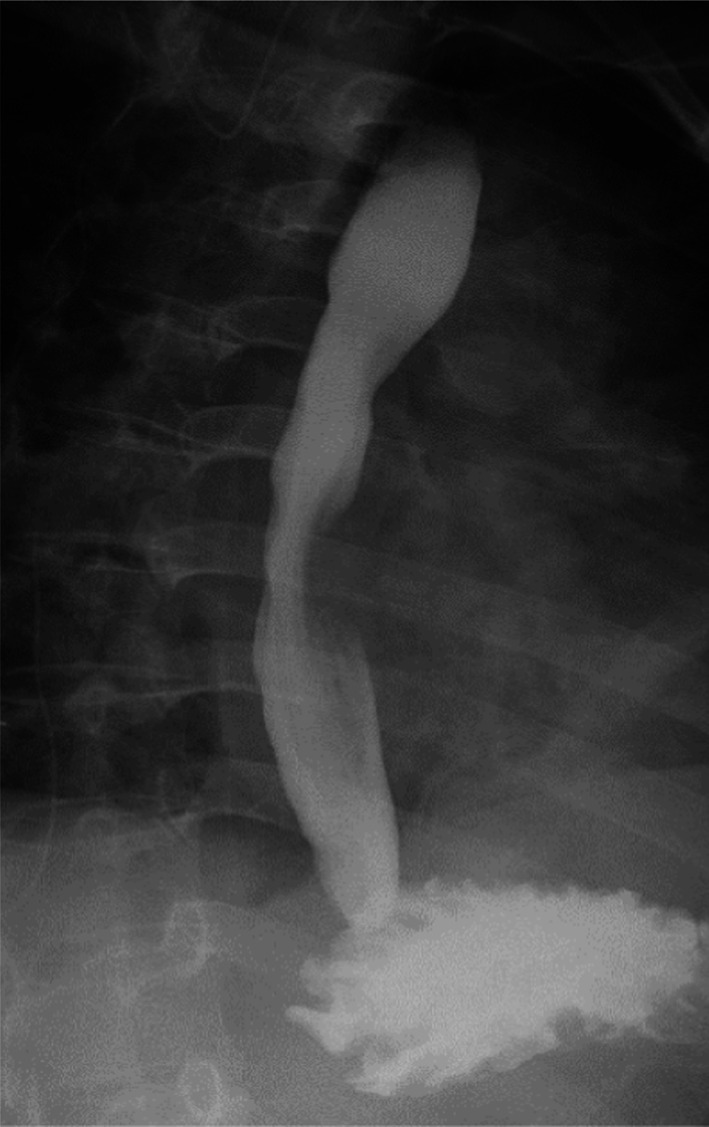
Postoperative esophagography on postoperative day 2

We initially exfoliated the azygos vein, double ligated it using silk, and double clipped both sides. In addition, a threaded needle was sewn through the dorsal blood vessel stump, grasped with an Endo Close® suturing device and pulled out the dorsal side, after which the suture was pulled back to expand the surgical field. We then opened the superior mediastinal pleura cranially and caudally from arch of the azygos vein and expanded the superior mediastinum. The upper thoracic esophagus was exposed, and the location of the tumor was roughly confirmed. A gastrointestinal endoscope was inserted to confirm the anal side of the tumor. An incision was made in the muscular layer of the upper thoracic esophagus with a monopolar hook and monopolar scissors, and the tumor was enucleated in a protective manner so as not to damage its capsule or mucosa (Additional file 1: Surgical Video). We applied appropriate robot-specific counter traction, changed the location of the traction frequently and, when necessary, increased it gradually and carefully. Moreover, to avoid any potential damage to the esophageal mucosa resulting from movement of the surgical field due to the patient’s heartbeat or respiration, a monopolar hook was inserted into the detached surface and a coagulation incision was made in a direction away from the mucosal side. The lobular mass on the anal side of tumor was enucleated with particular care. We put the tumor in a retrieval bag and removed it through an assist port, which was extended to match the tumor size. Air was then supplied from the gastrointestinal endoscope, and it was confirmed that there was no mucosal damage. We sewed up the muscularis using 4–0 Vicryl, inserting the endoscope in the thoracic esophagus, where it substituted for a bougie. In addition, the pleura was sutured using barbed suture (V-Loc®). We inserted a thoracic drain and had a closed wound. The operation time was 5 h and 29 min, and the hemorrhage volume was 21 ml. Postoperative esophagography on postoperative day 2 revealed that there was no leak or passage disorder (Fig. 5). The patient was discharged on postoperative day 4.

**Fig. 6:**
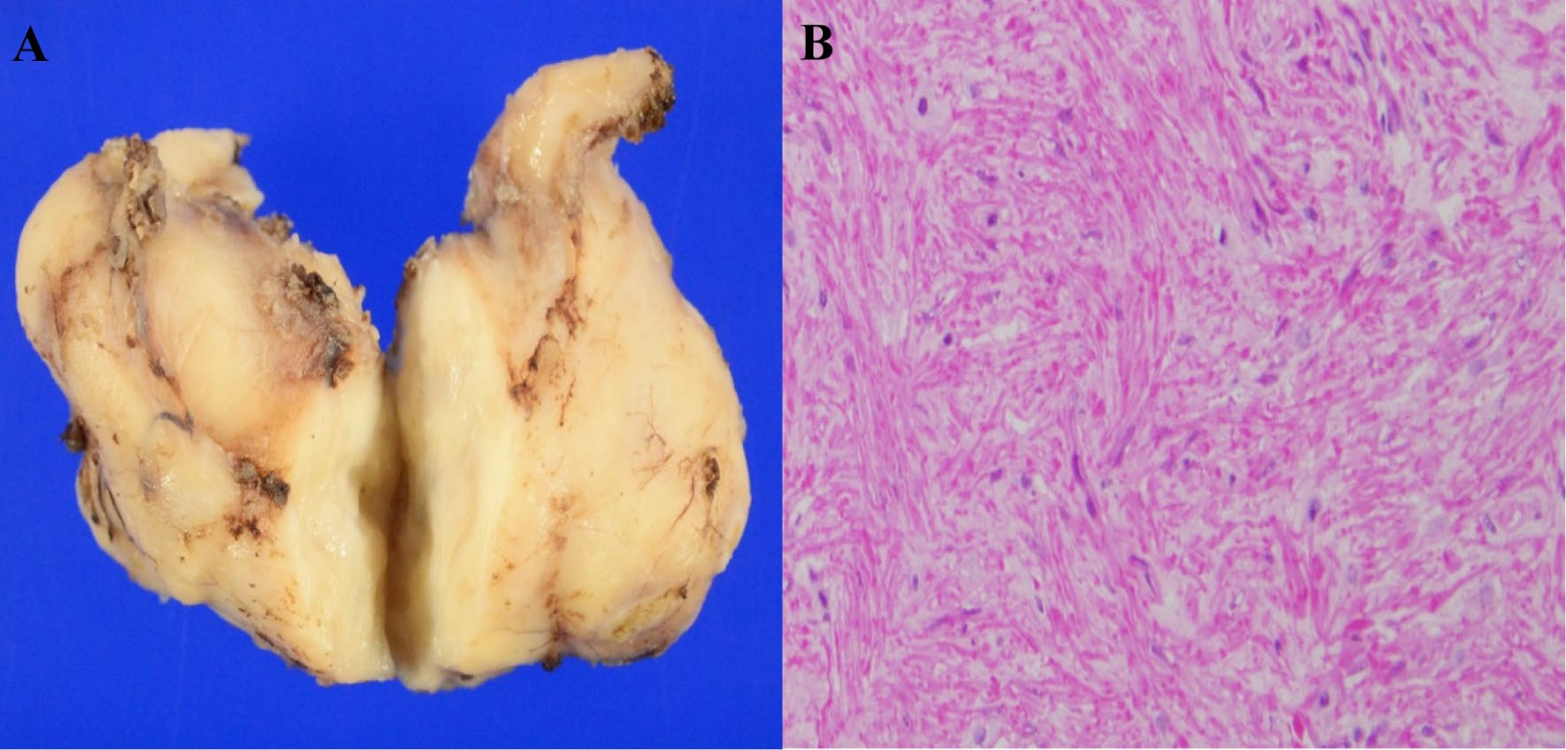
Appearance of the resected tumor (**A**). Hematoxylin–eosin staining shows proliferation of spindle-shaped tumor cells forming an interlacing pattern and fascicles (**B**)

The resected specimen was a white tumor 58 mm × 35 mm × 25 mm in size. There thus appears to have been shrinkage after excision (Fig. 6a). Hematoxylin–eosin staining showed proliferation of spindle-shaped tumor cells forming an interlacing pattern and fascicles (Fig. 6b), and immunostaining showed positivity for α-smooth muscle actin (α-SMA). Based on these findings a diagnosis of the esophageal leiomyoma was made.

## Discussion

Surgical treatment of esophageal leiomyoma is performed for symptomatic cases and for asymptomatic cases when there is evidence suggestive of malignancy or increasing tumor size. However, the indication for surgical treatment of esophageal leiomyoma is not yet established [4]. It is, therefore, necessary to design an acceptable treatment strategy for each patient. Although the use of VATS for enucleation of esophageal leiomyomas has increased in recent years, its two-dimensional vision and limited range of motion under the thoracoscope would make it difficult to completely enucleate a large tumor located in the upper mediastinum without disrupting mucosal integrity. Use of RATS for esophageal leiomyoma is not yet widespread, though there have been several reports of its use for that purpose [2, 3, 5–12]. The robotic approach offers advantages over VATS systems, including movement of the instruments in a manner that approximates the human wrist, three-dimensional vision, and ergonomic comfort for the surgeon. For esophageal surgery, which is delicate and within a confined space, where range of movement is limited, robot support is useful. In our case, the tumor was located in the upper-middle mediastinum, a position that made performance of the surgery difficult due to the very narrow field. We, therefore, thought that RATS could be very effective in this case. Based on earlier reports, use of RATS decreases the occurrence of surgical complications and the duration of the hospital stay as compared to VATS [2, 3, 5–32] (Table 1). It should be emphasized that there have been zero complications with RATS. On the other hand, there is no sense of touch with RATS, and it would not seem to be appropriate for enucleation of a relatively soft tumor. However, the three-dimensional vision provided by RATS is sufficient to greatly facilitate the approach to the tumor, despite the lack of a sense of touch. In addition, the wrist-like mobility of the robot appliance makes repair of the muscularis incision easier, and we can perform the suturation more exactly with no loss of technical precision or time. The same features enable treatment of injuries to the esophageal mucosa. For resection of an esophageal leiomyoma, the use of RATS has yielded a significant reduction in the incidence of mucosal injury, from 1 to 15% with both open thoracoscopy and laparotomy to 0% with RATS. Moreover, we were able to shorten the patients’ hospital stay and there were no serious complications with our two cases. Unfortunately, although it is clearly useful for these situations, the use of RATS for treatment of benign esophageal tumors and other disorders is not covered by medical insurance in Japan, and so it increases the cost of the operation.

**Table 1:** Surgical outcomes in between robot- and video-assisted thoracoscopic enucleation of esophageal leiomyoma reported between 2004 and 2021

Surgical approach	Author	Number of case	Operation time (minute)	Postoperative hospital stay (day)	Surgical complications (number of case)
RATS	Elli et al. [5]	2	120*	–	None
	Bodner et al. [6]	1	147	7	None
	Augustin et al. [7]	1	147	4	None
	DeUgarte et al. [8]	1	–	4	None
	Kernstine et al. [9]	1	104	1	None
	Ka-fung chiu et al. [10]	1	–	6	None
	Khalaileh et al. [2]	1	288	3	None
	Compean et al. [3]	1	–	3	None
	Inderhees et al. [11]	1	143	5	None
	Elliott et al. [12]	1	–	2	None
VATS	Akaraviputh et al. [13]	1	120	6	None
	Zaninotto et al. [14]	7	142*	6*	None
	Nguyen et al. [15]	3	190*	6*	None
	Dapri et al. [16]	1	85	3	None
	Saleh et al. [17]	1	–	2	None
	Luh et al. [18]	12	95*	7*	None
	Claus et al. [19]	10	89*	3*	None
	Hu et al. [20]	1	180	10	Mucosal injury(1)
	Macke et al. [21]	1	–	3	None
	Jeon et al. [22]	17	170*	4*	Mucosal injury(3)
	Kang et al. [23]	39	120*	9*	Mucosal injury (1)
	Maki et al. [24]	1	–	12	None
	Mujawar et al. [25]	1	–	6	None
	Zhang et al. [26]	8	60*	6*	None
	Chan et al. [27]	1	–	3	None
	Chen et al. [28]	1	–	8	None
	Li et al. [29]	52	123*	10*	Fistula (3)
	Alsinan et al. [30]	1	–	3	None
	Higuchi et al. [31]	1	288	7	None
	Milito et al. [32]	15	134*	4*	Atelectasis (1), Trocar site pain (1)

RATS, robot-assisted thoracoscopic surgery; VATS, video-assisted thoracoscopic surgery; *mean value

## Conclusion

Use of RATS for esophageal SMT enucleation is thought to enable more precise and delicate surgery without surgical complications, though various challenges remain to be overcome.

## Supplementary Information

**Additional file 1. ** Surgical Video.

## Data Availability

All data generated or analyzed during this study are included in this published article.

## Abbreviations

RATS: Robot-assisted thoracoscopic surgery
VATS: Video-assisted thoracoscopic surgery
SMT: Submucosal tumor
AL: Axillary line
ICS: Intercostal space
POD: Postoperative day
